# Hand function assessment in patients with juvenile idiopathic arthritis: usefulness of duruöz hand index

**DOI:** 10.1186/1546-0096-12-S1-P202

**Published:** 2014-09-17

**Authors:** Erbil Ünsal, Balahan Makay, Tuncay Duruöz

**Affiliations:** 1 Pediatric Rheumatology, Dokuz Eylül University Hospital, İzmir, Turkey; 2 Physical Medicine and Rehabilitation, Celal Bayar University Hospital, Manisa, Turkey

## Introduction

Assessing hand function in juvenile idiopathic arthritis (JIA) is important.

## Objectives

To assess if Duruöz’s Hand Index (DHI) was a useful instrument to evaluate functional disability in patients with JIA.

## Methods

Patients diagnosed as JIA according to ILAR criteria in Pediatric Rheumatology Department of Dokuz Eylül University Hospital were consecutively enrolled in this cross-sectional study. Demographic, clinical and functional characteristics of patients were evaluated. Erythrocyte sedimentation rate, patients’ and physicians’ global VAS, number of involved joints were recorded as non-functional parameters related to active disease status. Functional assessment was performed by DHI, Childhood Health Assessment Questionnaire (CHAQ), Purdue Pegboard, grip strength and 3 kinds of pinch strengths. The correlation of DHI was assessed with the other functional parameters as well as non-functional parameters by Spearman’s test. We also compared the functional parameters of patients with hand involvement with the ones who did not have involvement. The study protocol was approved by the institutional ethics committee and informed contents were obtained.

## Results

Forty JIA patients with a mean age of 12.3 ± 3.1 were recruited. Twenty seven of them (68%) were females. The average duration of disease was 3.4 ± 2.7 years (min: 0.5 max: 9 years). Twenty patients (50 %) had hand involvement. DHI was significantly correlated with CHAQ scores (rho=0.576, p<0.005), grip strength (Dominant hand: rho=0.399, p=0.011 and non-dominant hand: rho=0.391, p=0.013), patients’ global VAS (rho=0.452, p=0.004), physicians’ global VAS (rho=0.493, p=0.001), ESR (rho=0.456, p=0.004), number of involved joints (rho=0.487, p=0.002) and hand involvement (rho=0.580, p<0.005). DHI was not significantly correlated with Purdue Pegboard scores and 3 types of pinch strengths (p>0.05). The patients with hand involvement had significantly higher DHI scores than the patients without (0<0.005). However, other functional scores did not differ significantly between 2 groups (p>0.05). Besides, the mean ESR, physicians’ global VAS and CHAQ score were significantly higher in patients with hand involvement than the patients without (p=0.014, 0.001 and 0.044, respectively).

## Conclusion

The results of this study indicate that children with active disease have a greater risk of developing muscle weakness in the hands during the course of the disease. Hand strength in patients with JIA is mostly attributable to the activity of disease rather than hand involvement. DHI scores confirm these findings.

## Disclosure of interest

None declared.

